# Morbidity and long-term survival in patients with cervical re-exploration for papillary thyroid carcinoma

**DOI:** 10.1515/iss-2018-0023

**Published:** 2019-03-27

**Authors:** Nenia Baerbock, Anke Mittelstädt, Joachim Jähne

**Affiliations:** Clinic of Anaesthesiology and Intensive Care, Medizinische Hochschule Hannover, Carl-Neuberg-Str. 1, 30625 Hannover, Germany; Clinic for General and Digestive Surgery, Center for Endocrine, Oncologic and Metabolic Surgery, DIAKOVERE Henriettenstift, Hannover, Germany

**Keywords:** hypocalcemia, prognosis, recurrent laryngeal nerve paralysis, two-stage resections

## Abstract

**Background:**

Papillary thyroid carcinoma (PTC) has a favorable prognosis following one-stage surgical therapy, whereas two-stage resections bear the risk of increased morbidity and possibly impaired prognosis. To further elucidate the value of surgical re-exploration in PTC, a retrospective study was performed.

**Methods:**

The study involved 187 patients with PTC who underwent total thyroidectomy with central lymph node dissection between 2001 and 2011. The number of two-stage surgeries, the rates of recurrent laryngeal nerve paralysis (RLNP) as well as hypocalcemia, and the long-term survival were assessed.

**Results:**

Two-stage surgeries were performed in 43%. No statistically significant difference was seen between the one- and two-stage resection groups regarding the rate of RLNP (transient 5.6% vs. 6.3%, permanent 2.6% vs. 0%) nor for hypocalcemia (transient 25.2% vs. 18.8%, permanent 14.0% vs. 22.5%). The 10-year recurrence-free survival was 95.5% and the 10-year disease-specific survival was 98.9% with no difference between groups.

**Conclusion:**

Even though two-stage surgeries do not lead to a higher incidence of RLNP and hypocalcemia, optimal preoperative and intraoperative diagnostics have to be carried out to reduce the amount of completion surgeries.

**Abbreviations:** FNA, fine-needle aspiration; PTC, papillary thyroid carcinoma; RLNP, recurrent laryngeal nerve paralysis.

## Introduction

Thyroid cancer is a rare malignancy with an incidence of approximately 1% of all cancer entities [1]. This cancer includes a histological heterogeneous group subdivided by morphology. Papillary thyroid cancer (PTC) is the most common histological subtype with an excellent prognosis [1], [2], [3], [4]. The treatment of PTC includes a multimodal therapy in which surgery is the crucial part of the initial management. The aim of thyroidectomy and lymphadenectomy is minimal morbidity and mortality, on the one hand, and providing an excellent long-term prognosis, on the other hand. A high number of one-stage surgeries is considered to be a quality indicator in thyroid carcinoma surgery [1]. However, it has to be considered that because PTC is often diagnosed postoperatively, two-stage surgery as a surgical re-exploration is still common [1], [5], [6]. Moreover, two-stage surgery may be associated with a higher rate of postoperative morbidity [5], [7], [8], [9].

To further study the impact of two-stage surgery in the treatment of PTC and in order to add information to the already existing evidence, we performed a retrospective study to determine the factors that had an influence on two-stage surgery as well as morbidity and long-term survival.

## Materials and methods

The study included 187 patients with PTC undergoing total thyroidectomy with central lymph node dissection. All patients were treated in the Clinic for General and Digestive Surgery of the Diakovere Henriettenstift between January 1, 2001, and December 31, 2011. Patient data were anonymized. Preoperative examination and all clinical and histopathological records were obtained and stored in a database. Follow-up data were collected in close cooperation with the general practitioner and/or other medical institutions involved in the patients’ treatment.

The surgical procedure was performed according to the valid national guidelines at the time of surgery [10], [11], [12]. Microdissection technique and intermittent intraoperative nerve monitoring were applied in all cases. Primary surgery included all patients independent of the extent of resection. Completion surgery was defined as reoperation with the intention of total thyroidectomy and central lymph node dissection. Primary surgery with total thyroidectomy and central lymph node dissection was termed one-stage surgery. Two-stage surgeries included a primary surgery and a completion surgery. Completion surgery included thyroidectomy after initial partial resection, and/or lymph node dissection, the latter one in patients who had thyroidectomy at the initial operation.

All clinical and pathological reports were reviewed and assessed according to the categories of the TNM (tumor, node, metastasis) classification of 2010 [13]. Recurrent laryngeal nerve paralysis (RLNP) that did not regain normal vocal cord motility within 6 months after surgery was defined as permanent. Patients who showed symptoms of hypocalcemia and also had decreased serum calcium postoperatively were treated with calcium and vitamin D supplementations. Permanent hypocalcemia was defined as low serum calcium and low parathyroid hormone concentration, and either symptoms of hypocalcemia or calcium and/or vitamin D supplementation for >6 months. New local tumor manifestation or lymph node metastases that occurred 180 days after surgery and distant metastases that occurred 30 days after resection were defined as tumor recurrence.

Microsoft Excel (Microsoft Corporation, Redmond, WA, USA) and IBM SPSS Statistics 21 (IBM, Armonk, NY, USA) were used for data collection and statistical analysis. Besides the descriptive data analysis of the nominal and ordinal quantities, the chi-square-test as well as Fisher’s exact t-test were used to compare the differences between qualitative variables. The α for significance was set at p<0.05. In case of the transient and permanent RLNP, Fisher’s exact t-test was not applied due to the low number of events.

## Results

Of the 187 patients with PTC, most patients were female (78%, n=146) and the mean age was 50.3 years at the time of surgery (standard deviation ±15.9).

### Preoperative diagnostic work-up and intraoperative frozen sections

**Figure 1:**
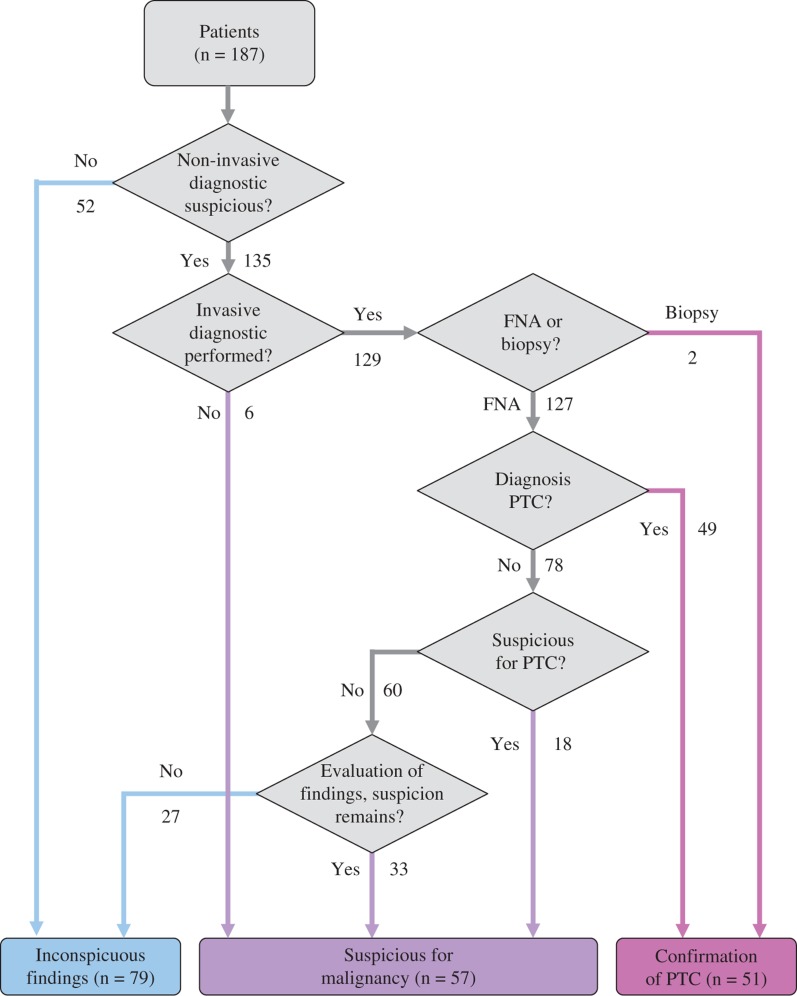
Preoperative diagnostic work-up (n=187).

If the non-invasive preoperative diagnostic work-up was suspicious for malignancy, fine-needle aspiration (FNA) cytology was mostly performed. A total of 127 patients (68%) underwent FNA cytology (Figure 1). The detailed results were as follows: 39% (n=49) malignant, 14% (n=18) suspicious for malignancy, 19% (n=24) follicular neoplasm, 4% (n=5) cell atypia of undetermined significance, and 24% (n=31) benign.

Overall, an intraoperative frozen section was carried out in 42% (n=79) of the patients. In 66 cases, the frozen section examination yielded a true-positive result with a sensitivity of 84%. The confirmation of PTC was made preoperatively in 27% (n=51), intraoperatively in 32% (n=59), and postoperatively in 41% (n=77) of the cases. In patients with suspicious FNA, 86% had intraoperative frozen sections (n=49).

### Surgical data

In 57% (n=107) of the patients, total thyroidectomy and central lymph node dissection (compartment Ia/Ib) was accomplished in one-stage surgery. In 80 patients (43%), completion surgery was indicated due to an incomplete resection in the primary surgery as well as an incidental postoperative finding of PTC. Table 1 shows the extent of two-stage surgery, which was done within 3 days after the initial surgical therapy in most cases (n=65, 81%).

**Table 1:** Extent of resection in two-stage surgery for PTC (n=80).

Extent of resection	Patients	
	n	%
Lymph node dissection compartment I	45	56.3
Completion thyroidectomy and lymph node dissection compartment I	34	42.5
Total thyroidectomy with lymph node dissection compartment Ib	1	1.3

### Postoperative morbidity

**Figure 2:**
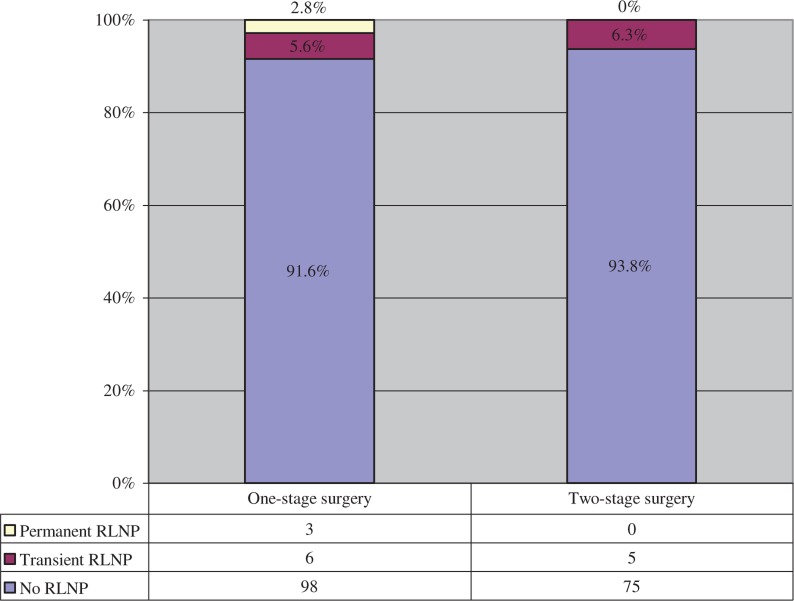
RNLP after one-stage (n=107) and two-stage (n=80) surgery.

Postoperative unilateral RNLP was observed in 14 patients (7.5%), of whom 11 patients (5.9%) had a transient RLNP and 3 patients (1.6%) had a permanent RLNP. The rate of postoperative RLNP was slightly lower in the group with two-stage surgery, but without any statistical significance. Within the one-stage group, three patients had a permanent RLNP, whereas in the two-stage group all of the patients recovered (Figure 2).

**Figure 3:**
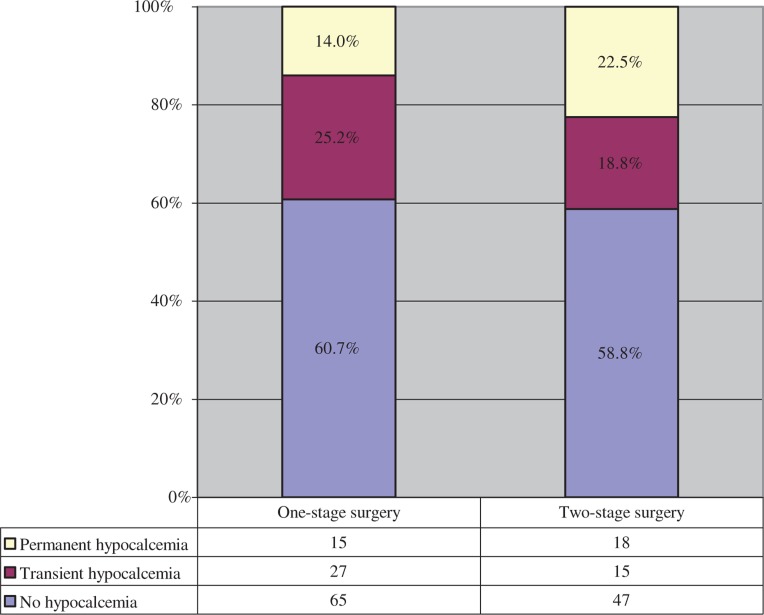
Hypocalcemia after one-stage (n=107) and two-stage (n=80) surgery.

Transient hypocalcemia was observed in 22.5% of the patients (n=42), and in 33 cases (17.6% of all patients) hypocalcemia remained permanent. The rates of transient and permanent hypocalcemia were lower in the one-stage group, again without any statistical significance (Figure 3).

### Pathological examination

Table 2 shows the pathological classification of PTC. More than half of the cases were classified as a tumor stage T3, and in 79.1% of the cases the tumor was unifocal. Positive lymph nodes were found in 61 patients (32.6%), and 3 patients had distant metastases.

**Table 2:** Pathological characteristics of PTC (n=187).

	PTC (n=187)		PTC_>10 mm_ (n=146)		PTC_≤10 mm_ (n=41)	
	n	%	n	%	n	%
Tumor stage						
T1a	16	8.6			16	39.0
T1b	36	19.3	36	24.7		
T2	28	15.0	28	19.2		
T3	101	54.0	76	52.1	25	61.0
T4a	6	3.2	6	4.1		
Focality						
Unifocal	148	79.1	123	84.2	25	61.0
Multifocal (unilateral)	12	6.4	5	3.4	7	17.1
Multifocal (bilateral)	27	14.4	18	12.3	9	22.0
Lymph node metastases						
N0	126	67.4	97	66.4	29	70.7
N1	61	32.6	49	33.6	12	29.3
Distant metastases						
M0	184	98.4	143	97.9	41	100
M1	3	1.6	3	2.1	0	0
Stage						
I	94	50.3	76	52.1	18	43.9
II	9	4.8	9	6.2		
III	76	40.6	53	36.3	23	56.1
IVa	5	2.7	5	3.4		
IVc	3	1.6	3	2.1		

### Follow-up and survival

**Figure 4:**
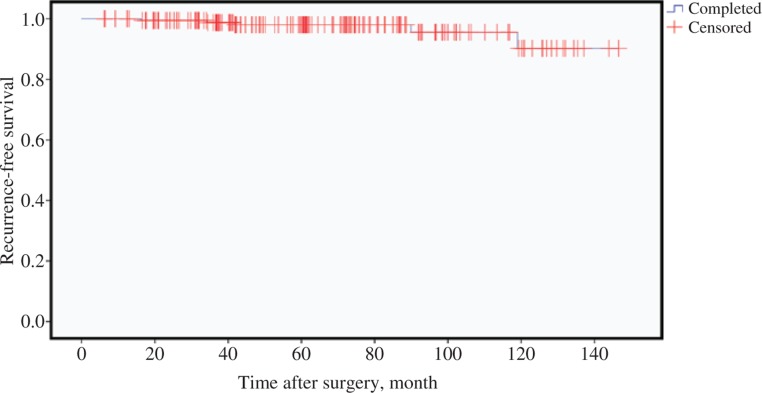
Recurrence-free survival (n=186).

The mean follow-up period was 71 months (range, 6–156 months). One patient was lost to follow-up; thus, the analysis of recurrence-free survival was based on 186 patients. In total, 165 patients (88%) received postoperative radioactive iodine therapy. Overall, five patients (3%) had a recurrence of the carcinoma and the 10-year recurrence-free survival was 95.5% (Figure 4). Three patients developed recurrence within the first 5 years, one patient after 7 years, and another patient shortly after 10 years.

**Figure 5:**
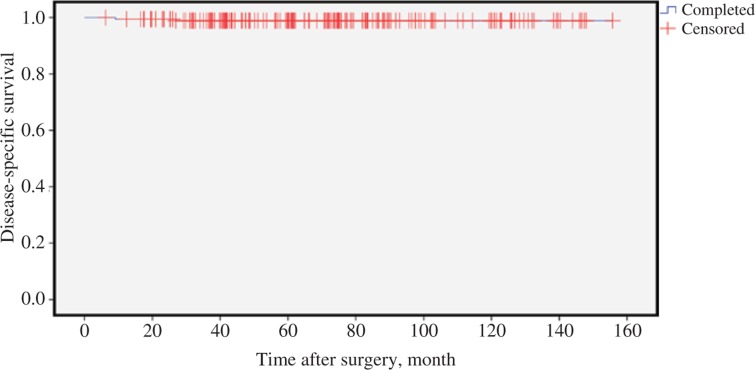
Disease-specific survival (n=187).

Nine of the 187 patients died within the observation period. Two patients died due to PTC, and in the other seven cases death was not tumor related. The 10-year disease-specific survival was 98.9% (Figure 5).

There were no differences in recurrence-free and disease-specific survival between the one-stage and two-stage surgical groups.

## Discussion

Thyroid carcinoma is often detected incidentally in the final histopathological examination [5]. In up to 15%, thyroid cancer is diagnosed in an assumed benign goiter [5], [6] and completion surgery is indicated in about 30% of these incidental findings [14]. In our study, the amount of two-stage total thyroidectomy with central lymph node dissection (43%) was relatively high compared to the data from the literature, in which a range from 5% to 50% is reported [6]. However, Dralle et al. [6] assumed that a theoretical value of just 10% is only achievable when optimal preoperative and intraoperative diagnostics are carried out. Furthermore, the composition of the study has an effect on the amount of two-stage surgeries. Our study only included patients with total thyroidectomy plus central lymph node dissection, and therefore a direct comparison to other studies is limited. Well-directed preoperative and intraoperative diagnostic strategies, in particular FNA and intraoperative frozen section, are helpful tools to minimize the amount of completion surgeries [1]. The diagnostic performance of FNA cytology in thyroid nodules is reported to have a sensitivity of 88–97% and a specificity of 47–98% [15]. The value of FNA cytology in our study was lower, with a false-negative rate of 24% and a true-positive rate of only 39%. On the contrary, Amrikachi et al. [16], whose study group like our study population included only patients with PTC, reported improved results with a false-negative rate of just 2% and a true-positive rate of 57%. The validity of the results is influenced by the position of the nodule, a correct aspiration and smear technique, as well as an adequate microscopic evaluation [3], [17]. Schmid [17] suggested that the process of smearing, preparation, and fixing should be transferred from the performing doctor to the cytopathologist. This process should be carried out in certified departments with at least 500 FNAs per year.

In the literature, the reliability of frozen section in thyroid nodules is reported with a sensitivity of 23–93% and a specificity of 91–100% [18]. In 42% of the patients in our study, frozen section was performed and the sensitivity was 84%. Yet, from these results, it cannot be concluded that the overall reliability of frozen section is as good as well. Therefore, the specificity is needed, which we could not define because our study did not include patients with a benign thyroid disease. The current “Practice guidelines for the surgical treatment of benign thyroid disease” [19] suggest performing frozen section examination in all patients with preoperative or intraoperative suspicion for malignancy. This should have been carried out in 57 patients of our study; however, it was carried out in only 49 cases (86%) without any identifiable reason. A reduction of two-stage surgeries can be achieved by improving the value of FNA cytology and a regular implementation of frozen sections. The current German guideline for the surgical management of malignant thyroid tumors recommends that a prophylactic central lymph node dissection should not be performed in a second surgery after initial total thyroidectomy in the primary surgery [3]. Consequently, the amount of two-stage surgeries may decrease.

RNLP and hypocalcemia are the most significant complications in thyroid surgery [2], [20]. The rates of RNLP range from 0% to 10% for transient paralysis and from 0% to 4% for permanent paralysis [21], [22], [23], [24], [25]. Our results with 5.9% transient paralysis and 1.6% permanent paralysis can be classified in the medium range. The transient and permanent hypocalcemia rates are reported to be from 7% to 40% and from 0% to 11% in the literature; in some studies, even higher rates are reported with up to 61% for transient and 16% for permanent hypocalcemia [21], [22], [23], [24], [25], [26], [27], [28]. Our transient rate of 23% was within the average, whereas the permanent rate of 18% was clearly in the upper range. The extent of surgery (total thyroidectomy and lymph node dissection), malignant thyroid disease, and two-stage surgery and reoperation are mainly seen as risk factors for both complications [1], [8], [9], [20], [23], [27]. However, our results showed that a two-stage total thyroidectomy with central lymph node dissection does not necessarily influence the complication rates, either for RNLP or for hypocalcemia. In detail, a reduced number of RLNP was observed in the two-stage surgery group. A statistical analysis was not possible because of the small number of events. The rate of postoperative hypocalcemia was nearly similar, although the rate of permanent hypocalcemia was descriptively higher in the two-stage surgery group, but without any statistically significant difference. Rafferty et al., Alvarado et al., and Ondik et al. [29], [30], [31] made similar observations. Rafferty et al. [29] showed that two-stage total thyroidectomy in comparison with one-stage surgery performed in patients with differentiated thyroid cancer does not pose a higher risk for both complications. Alvarado et al. and Ondik et al. [30], [31] came to the same conclusion in relation to two-stage central lymph node dissection in PTC. In contrast, other studies [8], [9], [20] deduced a clear correlation between a higher morbidity rate and two-stage surgery. The authors argued for strong adhesions and fibrinous inflammation in the operating field [7], [9]. Several studies analyzed the time interval between the primary surgery and the following completion surgery as a possible risk factor for the specific complications, and showed that the timing of the completion surgery has an important influence [7]. Glockzin et al. [5] verified and specified these results: surgery within 3 days or after 3 months reduces the risk of complications. Therefore, two-stage surgery is not inevitably associated with a higher complication rate [5], [14]. This might be an explanation for our results, because in the vast majority of the cases, the completion surgery was performed within the low-risk period.

A particular attention in the surgery of PTC is paid to central lymph node dissection, as it is mainly seen as a risk factor for the development of hypocalcemia [1], [22], [23], [27], [32]. Central lymph node dissection in patients with clinically proven lymph node metastases is clearly essential [3]. However, the extent of prophylactic lymph node dissection to gain a distinct advantage in the oncological benefit versus morbidity is still controversial [22], [23], [27], [32], [33], [34]. The current German guidelines [3] recommend that a prophylactic lymph node dissection should only be performed if the requisite surgical expertise is available. A prophylactic central lymph node dissection after total thyroidectomy is inadvisable for completion. In this line, the American Thyroid Association proposes in their guidelines [35] that prophylactic central lymph node dissection may not be performed for non-invasive T1 or T2 PTC. Recent studies support this approach by confirming that patients without prophylactic central lymph node dissection do not have a higher risk of recurrence but a reduced risk for surgical complications [22], [23], [32]. A comparison of our results in this context is not possible as our cohort only included surgeries with central lymph node dissection. Our results showed that five patients (3%) developed recurrence and two patients died because of PTC. The 10-year recurrence-free survival was 95.5%, and the disease-specific survival was 98.9%. According to the literature, which reports a 10-year recurrence-free survival of 80–97% and a 10-year disease specific-survival of 91–99% [4], [33], [36], [37], our data emphasize the good prognosis of PTC. Our high rate of hypocalcemia may correlate with central lymph node dissection. This hypothesis is supported by a study of Franzke et al. [38] in which postoperative hypoparathyroidism was analyzed in patients with surgery of benign goiter. The rate of postoperative hypocalcemia after total thyroidectomy was considerably smaller (21.7% vs. 40% in our current study). It should be noted that a part of the difference is due to the entity of thyroid disease (benign vs. malignant). Relating to the permanent rate of hypocalcemia, only an indirect comparison is possible because Franzke et al. did not report a separate rate for patients with total thyroidectomy. The rate of 0.37% also included patients with less than a total thyroidectomy; however, even under the assumption that all permanent hypocalcemias are a consequence of total thyroidectomy, the maximum rate sums up to 0.45%. Our comparable result of 18% was clearly higher. Therefore, it can be reasoned that at least a part of the 18% is based on central lymph node dissection. In consideration of the good survival rates, it may be conceivable that a less aggressive management is reasonable to avoid hypocalcemia.

## Conclusion

Although two-stage surgeries do not necessarily lead to a higher incidence of RLNP and hypocalcemia, optimal preoperative and intraoperative diagnostics should be carried out to reduce the amount of completion surgeries. Provided that the good survival rate will remain constant, it is conceivable that a less extent of surgery in selected patients is eligible to reduce the risk of hypocalcemia.

## Supplementary Material

The article (iss-2018-0023) offers reviewer assessments as supplementary material.
