# Supplementary material for: Morbidity and long-term survival in patients with cervical re-exploration for papillary thyroid carcinoma
Source: Innov Surg Sci. 2019 Mar 27;4(3):91–9. doi: 10.1515/iss-2018-0023 (PMC6817731; doi:10.1515/iss-2018-0023)
Supplement: Supplementary file 1 [file iss-4-20180023-s001.pdf]

Reviewer Assessment

Nenia Baerbock*, Anke Mittelstädt and Joachim Jähne

Morbidity and long-term survival in patients with cervical re-exploration for papillary thyroid carcinoma

https://doi.org/10.1515/iss-2018-0023
Received August 13, 2018; accepted March 5, 2019

***Corresponding author: Nenia Baerbock,** Clinic of Anaesthesiology and Intensive Care, Medizinische Hochschule Hannover, Carl-Neuberg-Str. 1, 30625 Hannover, Germany, E-mail: Baerbock.Nenia@mh-hannover.de

Reviewers' Comments to Original Submission

Reviewer 1: Martin Memming

Feb 19, 2019

**Reviewer Recommendation Term:**	Accept
**Overall Reviewer Manuscript Rating:**	N/A
**Custom Review Questions**	**Response**
Is the subject area appropriate for you?	5 - High/Yes
Does the title clearly reflect the paper's content?	5 - High/Yes
Does the abstract clearly reflect the paper's content?	5 - High/Yes
Do the keywords clearly reflect the paper's content?	5 - High/Yes
Does the introduction present the problem clearly?	5 - High/Yes
Are the results/conclusions justified?	5 - High/Yes
How comprehensive and up-to-date is the subject matter presented?	5 - High/Yes
How adequate is the data presentation?	5 - High/Yes
Are units and terminology used correctly?	5 - High/Yes
Is the number of cases adequate?	5 - High/Yes
Are the experimental methods/clinical studies adequate?	5 - High/Yes
Is the length appropriate in relation to the content?	5 - High/Yes
Does the reader get new insights from the article?	5 - High/Yes
Please rate the practical significance.	5 - High/Yes
Please rate the accuracy of methods.	5 - High/Yes
Please rate the statistical evaluation and quality control.	5 - High/Yes
Please rate the appropriateness of the figures and tables.	5 - High/Yes
Please rate the appropriateness of the references.	5 - High/Yes
Please evaluate the writing style and use of language.	5 - High/Yes
Please judge the overall scientific quality of the manuscript.	5 - High/Yes
Are you willing to review the revision of this manuscript?	Yes

Open Access. © 2019 Baerbock N., et al., published by De Gruyter. This work is licensed under the Creative Commons Attribution 4.0 Public License.

Comments to Authors:

The aim of this study was to elucidate the value of surgical re-exploration in patients with PTC, further the impact of two-stage surgery and to determine the factors which had an influence on two-stage surgery as well as morbidity and long-term survival.
In this one-center study is shown, that the situation and the surgical therapy of thyroid cancer does not always correspond to the data from the literature. The surgical results in this study are - especially to the group of two-stage surgeries - better than expected. Although the number of included patients is high enough to be convincing is it not for statistical significance. However, the results of all relevant parameters are showing, that in the morbidity and the long-term survival, against the common literature, there is no difference between the one-stage patients and the patients who underwent re-exploration. This is a strong indication for an excellent surgery and well done organization for re-exploration within not more than three days.
In the decade 2001 to 2011, when patients of this study underwent surgical therapy of PTC, it was clearly recommended to complete surgical therapy when PTC is diagnosed postoperatively with thyroidectomy and lymphadenectomy of the central compartments 1a and 1b. So there is no doubt to the surgical indication position.
The representation of reasons for lack of preoperative diagnosis (PTC) is not unusual but normally situation in thyroid surgery. This paper shows exactly the possibilities to handle this problem and it is discussed what can changed in future.
Naturally thyroid surgery in patients with PTC should be performed in one step. But as shown there are a lot of reasons that can prevent reaching this aim. Therefore this is an important study, that shows us the daily reality, the procedure and discussion in this center of thyroid surgery. Here are shown very good results in spite of adversities to perform every patient with PTC in one step.

Reviewer 2: Wolfgang Hiller

Oct 28, 2018

**Reviewer Recommendation Term:**	Accept with Minor Revision
**Overall Reviewer Manuscript Rating:**	70

**Custom Review Questions**	**Response**
Is the subject area appropriate for you?	4
Does the title clearly reflect the paper's content?	5 - High/Yes
Does the abstract clearly reflect the paper's content?	5 - High/Yes
Do the keywords clearly reflect the paper's content?	5 - High/Yes
Does the introduction present the problem clearly?	5 - High/Yes
Are the results/conclusions justified?	5 - High/Yes
How comprehensive and up-to-date is the subject matter presented?	5 - High/Yes
How adequate is the data presentation?	4
Are units and terminology used correctly?	4
Is the number of cases adequate?	5 - High/Yes
Are the experimental methods/clinical studies adequate?	5 - High/Yes
Is the length appropriate in relation to the content?	5 - High/Yes
Does the reader get new insights from the article?	3
Please rate the practical significance.	3
Please rate the accuracy of methods.	5 - High/Yes
Please rate the statistical evaluation and quality control.	4
Please rate the appropriateness of the figures and tables.	4
Please rate the appropriateness of the references.	5 - High/Yes
Please evaluate the writing style and use of language.	2
Please judge the overall scientific quality of the manuscript.	3
Are you willing to review the revision of this manuscript?	Yes

Comments to Authors:

Page 2 line 1:
When quoting the incidence of a disease the underlying population always has to be mentioned. In this case it refers to all patients with thyroid nodules (?)
Page 2 line 6: one side instead of on side
Material and Methods line 5: all instead of al
Page 3: Fisher (was an English scientist) instead of Fischer

The paper should be revised by a native English speaker
e.g.: „Table I shows the extent of two-stage surgery which was done within three days after initial surgical therapy in most cases“ instead of „Table I shows the extent of the two-stage surgery, which was on most cases done within three days after initial surgical therapy“
Page 4:
an incidental finding instead of an incidentally finding
two astage asurgery instead oit the two-stage surgery
Page 5:
Table 1 Extent instead of Extend
11 of whom instead of of whom 11 patients
...

Authors’ Response to Reviewer Comments

Oct 30, 2018

Paper has been revised especially in the English language. All changes are highlighted yellow. Coment 1 of Reviewer #2: Thyroid cancers are rare malignancies with an incidence of approximately 1% of all cancer entities.

Reviewers’ Comments to Revision

Reviewer 1: Wolfgang Hiller

Mar 03, 2019

**Reviewer Recommendation Term:**	Accept
**Overall Reviewer Manuscript Rating:**	80
**Custom Review Questions**	**Response**
Is the subject area appropriate for you?	5 - High/Yes
Does the title clearly reflect the paper’s content?	5 - High/Yes
Does the abstract clearly reflect the paper’s content?	5 - High/Yes
Do the keywords clearly reflect the paper’s content?	5 - High/Yes
Does the introduction present the problem clearly?	4
Are the results/conclusions justified?	4
How comprehensive and up-to-date is the subject matter presented?	5 - High/Yes
How adequate is the data presentation?	5 - High/Yes
Are units and terminology used correctly?	5 - High/Yes
Is the number of cases adequate?	5 - High/Yes
Are the experimental methods/clinical studies adequate?	4
Is the length appropriate in relation to the content?	4
Does the reader get new insights from the article?	4
Please rate the practical significance.	4
Please rate the accuracy of methods.	4
Please rate the statistical evaluation and quality control.	4
Please rate the appropriateness of the figures and tables.	4
Please rate the appropriateness of the references.	5 - High/Yes
Please evaluate the writing style and use of language.	3
Please judge the overall scientific quality of the manuscript.	4
Are you willing to review the revision of this manuscript?	Yes

Comments to Authors:
I have no further comments.
